# Localized In Vivo Prodrug Activation Using Radionuclides

**DOI:** 10.2967/jnumed.124.268559

**Published:** 2025-01

**Authors:** Jeremy M. Quintana, Fangchao Jiang, Mikyung Kang, Victor Valladolid Onecha, Arda Könik, Lei Qin, Victoria E. Rodriguez, Huiyu Hu, Nicholas Borges, Ishaan Khurana, Leou I. Banla, Mariane Le Fur, Peter Caravan, Jan Schuemann, Alejandro Bertolet, Ralph Weissleder, Miles A. Miller, Thomas S.C. Ng

**Affiliations:** 1 Center for Systems Biology, Massachusetts General Hospital, Boston, Massachusetts;; 2 Department of Radiation Oncology, Massachusetts General Hospital, Harvard Medical School, Boston, Massachusetts;; 3 Department of Imaging, Dana-Farber Cancer Institute, Harvard Medical School, Boston, Massachusetts;; 4 Office of Radiation Safety, Massachusetts General Hospital, Boston, Massachusetts;; 5 Department of Radiology, Massachusetts General Hospital, Harvard Medical School, Boston, Massachusetts;; 6 Institute for Innovation in Imaging, Massachusetts General Hospital, Boston, Massachusetts; and; 7 Department of Systems Biology, Harvard Medical School, Boston, Massachusetts

**Keywords:** theranostics, radionuclide therapy, combination chemoradiotherapy, FAPI

## Abstract

Radionuclides used for imaging and therapy can show high molecular specificity in the body with appropriate targeting ligands. We hypothesized that local energy delivered by molecularly targeted radionuclides could chemically activate prodrugs at disease sites while avoiding activation in off-target sites of toxicity. As proof of principle, we tested whether this strategy of radionuclide-induced drug engagement for release (RAiDER) could locally deliver combined radiation and chemotherapy to maximize tumor cytotoxicity while minimizing off-target exposure to activated chemotherapy. **Methods:** We screened the ability of radionuclides to chemically activate a model radiation-activated prodrug consisting of the microtubule-destabilizing monomethyl auristatin E (MMAE) caged by a radiation-responsive phenyl azide, and we interpreted experimental results using the radiobiology computational simulation suite TOPAS-nBio. RAiDER was evaluated in syngeneic mouse models of cancer using the fibroblast activation protein inhibitor (FAPI) agents [^99m^Tc]Tc-FAPI-34 and [^177^Lu]Lu-FAPI-04 and the prostate-specific membrane antigen (PSMA) agent [^177^Lu]Lu-PSMA-617, combined with caged MMAE or caged exatecan. Biodistribution in mice, combined with clinical dosimetry, estimated the relationship between radiopharmaceutical uptake in patients and anticipated concentrations of activated prodrug using RAiDER. **Results:** RAiDER efficiency varied by 70-fold across radionuclides (^99m^Tc > ^111^In > ^177^Lu > ^64^Cu > ^32^P > ^68^Ga > ^223^Ra > ^18^F), yielding up to 320 nM prodrug activation/Gy of exposure from ^99m^Tc. Computational simulations implicated low-energy electron–mediated free radical formation as driving prodrug activation. Radionuclide-activated caged MMAE restored the prodrug’s ability to destabilize microtubules and increased its cytotoxicity by up to 2,600-fold that of the nonactivated prodrug. Mice treated with [^99m^Tc]Tc-FAPI-34 and caged MMAE accumulated concentrations of activated MMAE that were up to 3,000 times greater in tumors than in other tissues. RAiDER with [^99m^Tc]Tc-FAPI-34 or [^177^Lu]Lu-FAPI-04 delayed tumor growth, whereas monotherapies did not (*P* < 0.003). Clinically guided dosimetry suggests sufficient radiation doses can be delivered to activate therapeutically meaningful levels of prodrug. **Conclusion:** This proof-of-concept study shows that RAiDER is compatible with multiple radionuclides commonly used in nuclear medicine and can potentially improve the efficacy of radiopharmaceutical therapies to treat cancer safely. RAiDER thus shows promise as an effective strategy to treat disseminated malignancies and broadens the capability of radiopharmaceuticals to trigger diverse biologic and therapeutic responses.

## Visual Abstract

**Figure:**
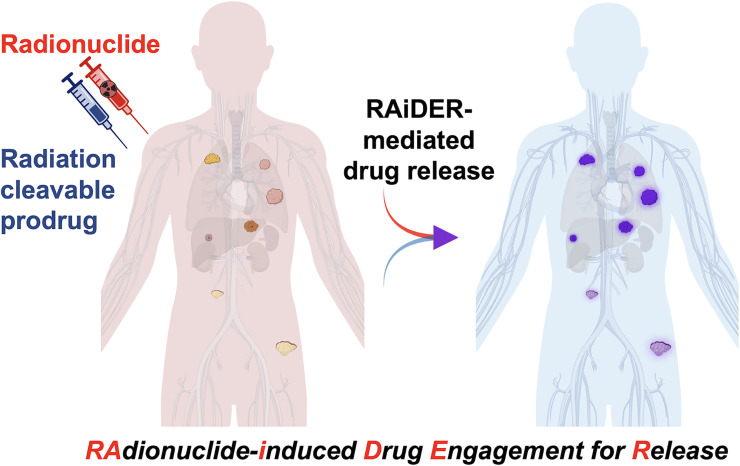

Radiopharmaceutical therapy (RPT) is becoming a critical pillar in cancer treatment, exemplified by U.S. Food and Drug Administration approvals of [^177^Lu]Lu-PSMA-617 (1) and [^177^Lu]Lu-DOTATATE (2) for managing advanced-stage prostate and neuroendocrine tumors. Encouraging phase III trials (NCT04689828 and NCT03972488) are poised to extend RPT indications to treatment settings earlier in the disease course (3). However, current RPT agents cannot mediate complete disease control in all patients and must be combined with other treatments to effectively manage advanced disease (4).

Systemic chemo- and immunotherapies are being actively tested with RPT. As standalone therapies, they form the mainstay of treatment in patients with disseminated disease (5). Although chemo- and immunotherapies are often effective for tumor control, dose-limiting toxicities to off-target body sites constrain therapeutic indices (6). In some cases, patients experience high-grade toxicities (7), exacerbated when multiple drugs are used in combination (8) or when other medical conditions exist. This results in dose reduction or treatment cessation, reducing efficacy and potentially contributing to treatment resistance (8). Therefore, the ability to selectively deliver and activate potent drugs in tumors while minimizing their impact elsewhere in the body would be highly desirable.

Numerous drug delivery strategies have been developed to expand the range over which therapeutic cargoes can safely elicit effective responses (9). Examples include antibody–drug conjugates and nanomedicines designed to improve pharmacokinetics and payload delivery to tumors (10), prodrugs that release activated drugs in the presence of enriched enzymes or hypoxia (11), intraarterial and direct tumor delivery, and strategies using externally delivered triggers such as ultrasound to control drug activity (12). Although such approaches can increase tumor target specificity and efficacy, unintended off-target activity remains a challenge. Inevitable accumulation in clearance tissues (13), heterogeneity of physiochemical properties within the tumor microenvironment, and premature drug activation because of chemical instability represent challenges for existing drug delivery approaches (14).

Ionizing radiation shows promise for triggering prodrug activation locally in tumors and addresses some limitations (15–17). In principle, radiation-labile caging moieties enable precise spatiotemporal chemical activation of otherwise inactive prodrugs using external beam irradiation. A benefit of this approach over other prodrug strategies is that activation is bioorthogonal, because drug release is triggered only by ionizing radiation. This approach can enhance the release of activated chemotherapy within the tumor and block tumor growth without detectable systemic toxicities (15,16,18).

Translation of this approach is attractive for locoregional or oligometastatic disease when using external beam radiation therapy (EBRT) but impractical for multifocal metastases (19). Use of radionuclides from RPT or other nuclear medicine examinations for prodrug activation would extend the radiation drug activation paradigm for treating disseminated disease. Furthermore, when combined with RPT, local drug activation could combine with the radiotoxic effects of the radionuclide, a potentially powerful synergy (3,4). Although radiolysis for maintaining radionuclide–conjugate stability has been explored (20), the application of a radionuclide-mediated drug activation approach remains limited (21).

The objective of this study is to assess the feasibility of the radionuclide-induced drug engagement for release (RAiDER) approach (Fig. 1) for prodrug activation using a recently developed chemical strategy for long-circulating radiation-labile drug conjugates.

**FIGURE 1.**
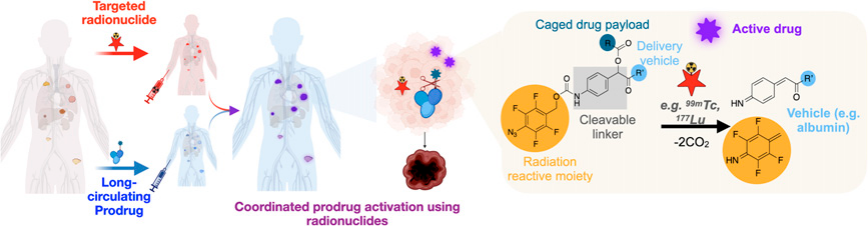
RAiDER concept. Targeted radionuclides accumulate in tumor tissues, locally delivering radiation to chemically activated caged prodrugs. Radionuclide-mediated prodrug activation occurs through reduction of phenyl azide caging moiety (orange), leading to linker self-immolation and release of active drug payload (purple) from its drug delivery vehicle (blue), which in this work is long-circulating serum albumin. Consequently, locally activated therapeutic payloads combine with ionizing radiation to maximize tumor cytotoxicity while sparing off-target tissues. Image created with BioRender.com.

## MATERIALS AND METHODS

Detailed experimental methods are presented in the supplemental materials (supplemental materials are available at http://jnm.snmjournals.org). All animal research was performed under guidelines and approval from the local Institutional Animal Care and Use Committee.

## RESULTS

### Synthesis and Chemical Characterization of Long-Circulating Radiation-Activated Prodrugs

RAiDER was developed using a recent design for long-circulating prodrugs (18). As proof of concept, therapeutic payloads consisting of monomethyl auristatin E (MMAE) or exatecan were conjugated to albumin via a radiation-labile para-azido-2,3,5,6-tetrafluorobenzyl moiety and self-immolating linker (albumin-conjugated caged MMAE or caged exatecan; Supplemental Schemes 1–3; Supplemental Figs. 1–9) (18). Constructs were stable in phosphate-buffered saline (pH 7.4) for 4 wk at 4°C (Supplemental Fig. 8).

### Select Radionuclide Types Can Efficiently Mediate RAiDER

**FIGURE 2.**
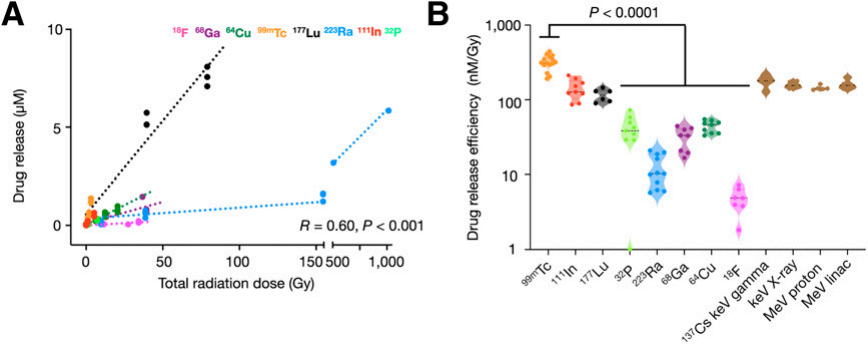
Radionuclide-mediated drug release from caged MMAE. (A) Caged MMAE (10 µM) was exposed to different radionuclides with varying activities. Corresponding total radiation dose release was estimated using TOPAS-nBio (26) and compared with prodrug-activated drug release, indicating varying radionuclide efficiencies (µM/Gy). (B) Drug release efficiencies (nM/Gy) across radionuclides and external beam modalities. Highest release efficiency (^99m^Tc) was compared with other nuclides or modalities (Violin plots: black line, median; dotted color lines, quartiles, 1-way ANOVA with Tukey multiple comparison test). Some values for ^137^Cs irradiation were previously presented (18) and reproduced here to facilitate comparison. Pearson correlation coefficient *R* was calculated across all data points. linac = linear particle accelerator.

The ability of commonly used clinical radionuclides to induce radiation-labile prodrug activation was evaluated in vitro. The α-emitters, electron–positron emitters, and γ-emitters were assessed across preclinical and clinically relevant activities (Fig. 2A; Supplemental Table 1). Results indicate roughly linear relationships between caged-MMAE activation and total dose, with variable drug release efficiency among radionuclides (Fig. 2B). ^99m^Tc, ^111^In, and ^177^Lu showed the highest efficiencies; ^99m^Tc was approximately 2-fold more efficient than EBRT, whereas ^111^In and ^177^Lu performed similarly. They were followed by other β- and α-emitting nuclides (^32^P, ^64^Cu, ^68^Ga, and ^223^Ra). Near-pure positron-emitting ^18^F was less efficient.

### In Vitro Cytotoxicity of Radionuclide-Released Prodrugs

**FIGURE 3.**
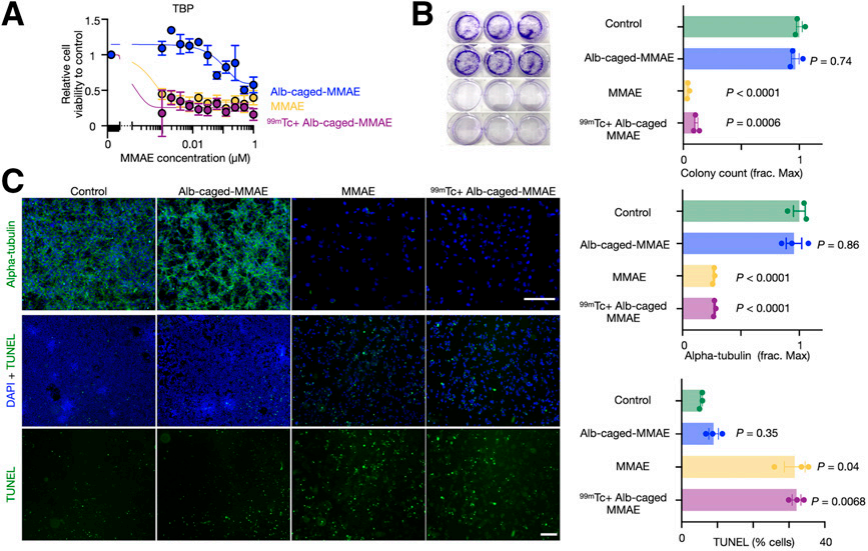
RAiDER restores biologic prodrug activities in vitro. (A) Cytotoxicity of albumin (Alb)–conjugated caged MMAE; ^99m^Tc-activated, Alb-conjugated caged MMAE; and free MMAE on TBP3743 anaplastic thyroid cancer, measured 72 h after treatment by resazurin-based assay (*n* = 3, mean ± SE). (B) Representative images and quantification of TBP3743 colony formation (mean ± SE, 2-way ANOVA with Geisser–Greenhouse correction). (C) Representative images of microtubule immunofluorescence (top) and apoptosis by terminal deoxynucleotidyl transferase-mediated dUTP nick-end labeling (TUNEL; bottom) 24 h after treatment in TBP3743 cells (mean ± SE; 2-way ANOVA with Geisser–Greenhouse correction; scale bars, 200 µm). DAPI = 4′,6-diamidino-2-phenylindole; frac. max = fraction of maximal count/signal as observed in control conditions.

Cytotoxicity of the radionuclide-released drug was tested in vitro across aggressive murine and human cancer models spanning colon (MC38), prostate (LNCaP), anaplastic thyroid (TBP3743), fibrosarcoma (HT1080), pancreatic (iKRAS), and ovarian cancers (BBPNM). Many of these diseases respond poorly to conventional chemotherapy (22). ^99m^Tc was used because of its high drug-activating efficiency. Albumin-conjugated caged-MMAE activation by ^99m^Tc elicited a more than 400-fold increase in drug toxicity for most models tested, as measured by a 72-h cell proliferation assay and similar to native MMAE (Fig. 3A; Supplemental Fig. 10; Supplemental Table 2). ^99m^Tc alone was not cytotoxic with activities used for drug activation (Supplemental Fig. 11). Clonogenic assays revealed that ^99m^Tc-activated, albumin-conjugated caged MMAE significantly blocked colony formation (Fig. 3B). Consistent with external irradiation (18), ^99m^Tc-activated, albumin-conjugated caged MMAE restored the ability of MMAE to destabilize cellular microtubules and induce apoptosis, as seen by immunofluorescence staining of α-tubulin and terminal deoxynucleotidyl transferase-mediated dUTP nick-end labeling, respectively (Fig. 3C).

### RAiDER Depends on Energy-Specific Free Radical Formation

Free radical radiolysis products, including superoxide, hydroxyl radicals, and hydrated electrons, underlie many radiation-dependent chemical reactions, including uncaging of para-azido-2,3,5,6-tetrafluorobenzyl and radiopharmaceutical stability (20,23–25). Therefore, we assessed whether radical scavengers could inhibit RAiDER and found that gentisic acid and ascorbic acid could reduce caged-MMAE prodrug activation by ^99m^Tc (Supplemental Fig. 12A), confirming that RAiDER depends on the availability of free radicals generated by ionizing radiation.

**FIGURE 4.**
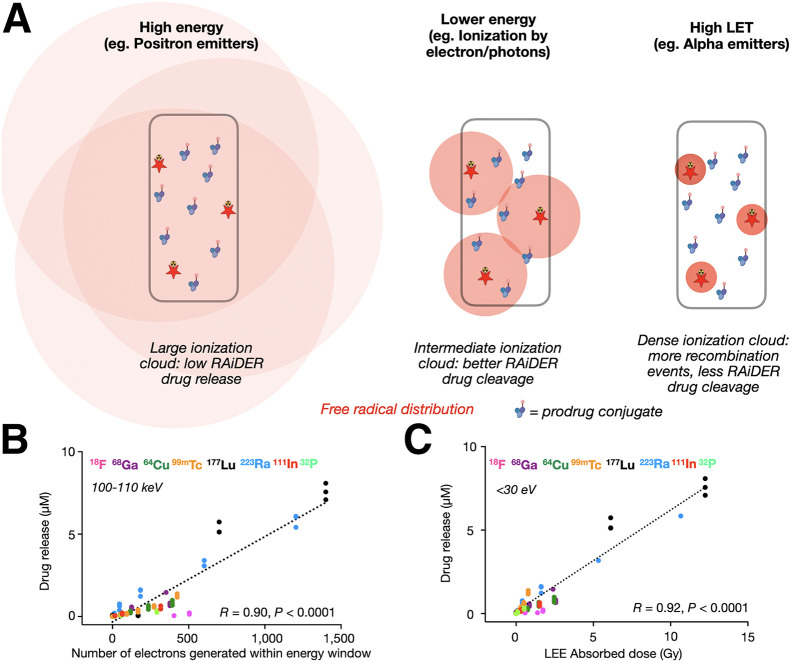
Computational modeling of radionuclide-mediated drug release using TOPAS-nBio. (A) Conceptual mechanism to explain radionuclide-dependent RAiDER efficiencies. Red shading illustrates spatial distribution and frequency of free radical–generating ionization events from given radionuclide within vial. Ionization clouds interact with prodrug molecules most efficiently for nuclides generating lower-energy electrons. (B) Correlation between observed drug release across radionuclides and their estimated number of electrons generated within 100- to 110-keV energy window. (C) Comparison of drug release efficiency with dose imparted by low-energy electrons (LEEs) across radionuclides. Pearson correlation coefficient *R* was calculated across all data points. LET = linear energy transfer.

Free radical generation from water radiolysis occurs by direct ionization from initial radionuclide decay events but more predominantly by indirect ionizations from secondary electron cascades with progressively lower energies along the ionization track (26,27). The spatial extent of these cascades depends on the initial energy of the electron from the original ionization. Radionuclides releasing high-energy particles, such as positrons from ^18^F, may generate ionization events over a broader volume at lower concentrations than nuclides generating lower-energy initial electrons (28). The higher concentration of ionization cascades for the latter may improve the probability of local reaction with macromolecules (29). These assertions (Fig. 4A) were tested using the radiobiology simulation platform TOPAS-nBio (26). Simulations examining the number of electrons generated by radionuclides across different energy windows showed the best correlation with observed drug release across all nuclides for 100- to 110-keV electrons (*R* = 0.90, *P* < 0.0001; Fig. 4B; Supplemental Fig. 12B). Because low-energy electrons (<30 eV) are implicated in localized free radical formation (26,30), we additionally simulated the dose deposited by low-energy electrons within nanoscopic volumes (∼10 nm to reflect the hydrodynamic radius of albumin-conjugated caged MMAE that would enable radicals to chemically interact with the prodrug), finding that the dose delivered by low-energy electrons at this scale correlated better with drug release than the total dose imparted across all radionuclides (*R* = 0.92 vs. 0.60, *P* < 0.0001; Fig. 4C).

We tested the hypothesis that lower-energy radiation enables more efficient drug release using external beam irradiation. We simulated the energy spectra of 2 low-energy anode–filter combinations (W-Al and W-Rh) using SpekPy to model mammographic x-rays (31). Spectra from both filters presented higher fluence at lower energies (≤60 keV) than irradiation at 320 keV (Supplemental Fig. 13A; Supplemental Table 3). This suggests that mammography-generated x-rays would lead to more efficient drug release and was indeed observed experimentally (*P* < 0.0001; Supplemental Fig. 13B).

### Radionuclide-Mediated Drug Release In Vivo

**FIGURE 5.**
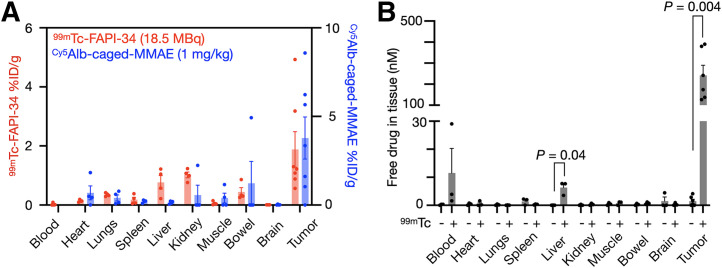
In vivo RAiDER biodistribution and prodrug activation. (A) Biodistribution of fluorescent albumin (Alb)–conjugated caged MMAE, measured by tissue Cy5 fluorescence, and [^99m^Tc]Tc-FAPI-34, measured by γ-scintillation counting in B6129SF1/J mice bearing syngeneic TBP3743 tumors. (B) Biodistribution of activated Alb-conjugated caged MMAE (with [^99m^Tc]Tc-FAPI-34) compared with nonactivated Alb-conjugated caged MMAE, as measured by liquid chromatography–mass spectrometry or high-performance liquid chromatography. Data are mean ± SE (*n* = 3–4 mice per condition, 2-tailed *t* test shown). %ID/g = percentage injected dose per gram of tissue.

The ability of RAiDER to mediate drug release in vivo was assessed using the TBP3743 syngeneic mouse model of anaplastic thyroid cancer (22). Immunofluorescence indicated that TBP3743 tumors express mouse fibroblast activation protein (FAP; Supplemental Fig. 14), and prior work suggests favorable tumor-to-background albumin uptake over 48 h (32). Therefore, we treated mice bearing TBP3743 tumors with Cy5-fluorescent albumin-conjugated caged MMAE, followed by [^99m^Tc]Tc-FAP inhibitor (FAPI)–34 (33,34) administration 48 h later and tissue harvesting 24 h later to assess biodistribution and prodrug activation (Fig. 5; Supplemental Figs. 15 and 16). This showed the highest accumulation and colocalization of both Cy5 fluorescence and [^99m^Tc]Tc-FAPI-34 in tumors. Other examined tissues showed uptake that was lower or discordant between the 2 agents. Selectivity for active MMAE accumulation was dramatically enhanced in tumors, 20–3,000 times more than in other tissues (Supplemental Fig. 17). Minimal caged-MMAE activation was seen without [^99m^Tc]Tc-FAPI-34 (Fig. 5). Estimated dosimetry shows that in vivo prodrug activation by [^99m^Tc]Tc-FAPI-34 was similar or higher than values observed in vitro (Supplemental Table 4) (35).

To evaluate the generalizability of RAiDER, we performed analogous biodistribution assays using a syngeneic murine model of prostate-specific membrane antigen (PSMA)–expressing prostate cancer (RM1.PSMA; Supplemental Fig. 18) by treating tumor-bearing mice with albumin-conjugated caged exatecan and [^177^Lu]Lu-PSMA-617 (Supplemental Fig. 18) (36). [^177^Lu]Lu-PSMA-617 accumulated in tumors and triggered the local accumulation of activated exatecan more selectively in tumors than when free exatecan was administered.

### RAiDER Improves Radionuclide Efficacy to Slow Tumor Progression

**FIGURE 6.**
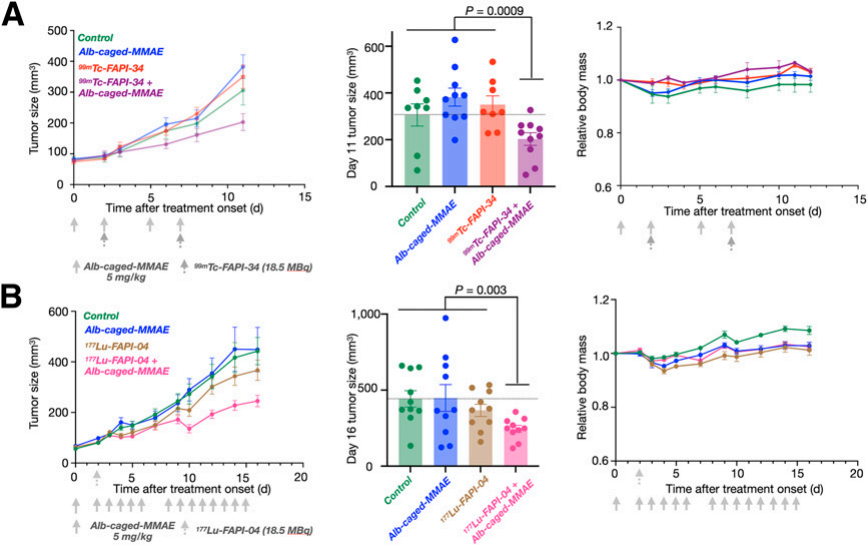
RAiDER enhances ability of targeted radionuclides to block tumor growth. (A and B) Mice bearing TBP3743 tumors were treated with albumin (Alb)–conjugated caged MMAE (5 mg/kg/dose) and either [^99m^Tc]Tc-FAPI-34 (A; 18.5 MBq/dose; *n* = 18 total mice, 36 total tumors) or [^177^Lu]Lu-FAPI-34 (B; 18.5 MBq; *n* = 20 total mice, 40 total tumors). Caliper measurements of tumor growth are shown over time (left) and with individual tumor sizes on indicated day (middle; 2-tailed Mann–Whitney tests compare combination and pooled control or monotherapies). Mouse mass was measured during treatment time course (right). Data are displayed as mean ± SE.

We next evaluated whether in vivo radionuclide-mediated prodrug activation could translate into detectable effects on disease progression. TBP3743 tumor-bearing mice were treated with albumin-conjugated caged MMAE, [^99m^Tc]Tc-FAPI-34, or their combination, and tumor growth was monitored. Tumor growth was delayed with combination treatment but not with monotherapy and control treatments (*P* = 0.0009; Fig. 6A), and 2-way ANOVA indicated prodrug and radiation synergy (2-way ANOVA interaction, *P* < 0.006). No noticeable toxicity was observed, as evidenced by mouse weight changes during treatment, blood biomarkers, and histology (Supplemental Figs. 19 and 20). Equimolar doses of free MMAE are known to be toxic in mice (18).

We additionally tested RAiDER with a single [^177^Lu]Lu-FAPI-04 (37) injection. RPT significantly delayed tumor growth (2-way ANOVA term for RPT, *P* < 0.02). This was more pronounced when combined with albumin-conjugated caged MMAE (Fig. 6B) than with control or monotherapy (*P* = 0.003), without detectable toxicity.

### Clinical Dosimetry Is Compatible with RAiDER

**FIGURE 7.**
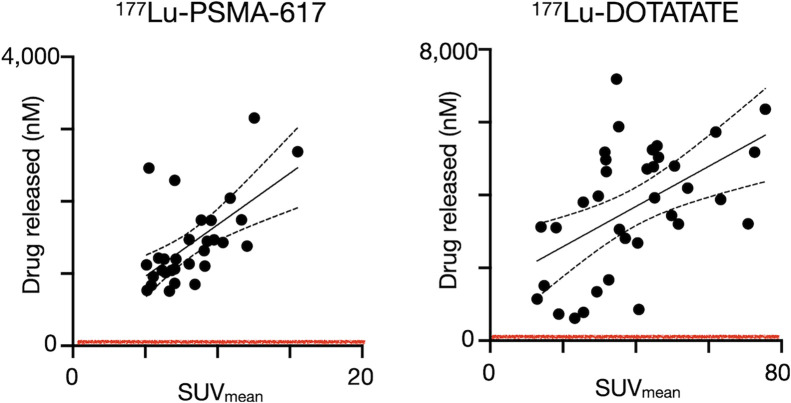
Estimating tumoral drug release in patients using clinical dosimetry. Lesion SUVs derived from [^68^Ga]Ga-PSMA PET and [^68^Ga]Ga-DOTATATE PET in prostate and neuroendocrine cancer patients receiving [^177^Lu]Lu-PSMA-617 (38) and [^177^Lu]Lu-DOTATATE (39), respectively, were compared with expected intratumoral active drug release (calculated based on release efficiencies in Fig. 2). Absorbed dose within each lesion was calculated in those previous studies using ^177^Lu SPECT/CT, demonstrating good correlation with PET SUV. Red line denotes inhibitory concentration of 50% estimated for aggressive cancers in this study (Supplemental Table 2).

The feasibility of RAiDER in patients was estimated using clinical dosimetry. Expected drug release in prostate and neuroendocrine tumors in patients treated with [^177^Lu]Lu-PSMA-617 and [^177^Lu]Lu-DOTATATE, respectively, was estimated with literature-based tumor dosimetry (38,39) using the drug activation efficiencies shown in Figure 2. Tumor accumulation of caged prodrugs was assumed to not be rate-limiting because of its observed biodistribution (Fig. 7) and pharmacokinetic modeling (18). Dosimetric analysis showed that radiopharmaceutical uptake in all lesions examined would be sufficient to mediate drug release well above the reported in vitro inhibitory concentration of 50% of MMAE that blocks cancer cell proliferation (Supplemental Table 2). Moreover, uptake in these lesions is well within the typical ranges seen in patients receiving RPT.

Extrapolation of these observations to other radiopharmaceuticals was assessed using MIRDcalc (40) and published pharmacokinetic data of agents in human use (41–43). As expected, the α-emitting isotopes with high linear energy transfer delivered the highest tumor dose per unit of activity, regardless of the delivery vehicle (2-way ANOVA with Tukey multiple comparison correction, *P* < 0.02; Supplemental Fig. 21; Supplemental Table 5). Scaling these values for clinically used activities (α, 20 MBq; β, 7,400 MBq; γ, 1,110 MBq), followed by an estimation of drug release based on the tumor dose delivered with these activities, shows that all classes of radionuclides, and especially ^177^Lu, can mediate drug release to enact meaningful cytotoxicity (at least an inhibitory concentration of 50%) across a range of tumor sizes and compositions. This analysis suggests that RAiDER may be appropriate across various clinical applications, including for patients with borderline RPT lesion uptake who may be less responsive to mono-RPT.

## DISCUSSION

This report presents RAiDER as a novel method to harness ionizing radiation imparted by radionuclides not only for their radiotoxic effects on tumors but also for mediation of the localized release of complementary molecular therapy. Timed coadministration of caged-drug payload (albumin-conjugated caged MMAE) and radiopharmaceutical enabled tumor colocalization of the 2 agents, enhanced drug release in tumors, and reduced drug release by 20–3,000 times in off-target tissues. Preclinical studies showed improved tumor control with RAiDER over mono-RPT without appreciable systemic toxicity.

Albumin-bound radiation-activated prodrugs were used in this proof-of-principle demonstration, because serum albumin circulates for an extended period in the body via FcRn-mediated recycling. Its 66.5-kDa molecular weight makes it smaller than antibodies and nanoparticles and promotes its ability to penetrate tissues and accumulate in cancer cells and phagocytes across multiple tumor types (18), facilitating translation of this approach across different malignancies. Para-azido-2,3,5,6-tetrafluorobenzyl and alternative radiation-labile chemistries have been conjugated with other macromolecules or drug delivery vehicles, including nanoparticles or antibodies (16,18), likely applicable to RAiDER. Because the phenyl azide caging moiety is less sensitive to positron emitters, as shown in this study, imaging versions of prodrugs could be synthesized to provide a companion imaging theranostic (3).

Ionizing radiation activates drugs in a process dependent on the generation and availability of reactive radical species. A key difference between RPT-based prodrug activation (RAiDER) and EBRT is that EBRT is delivered at a high dose rate across discrete fractionations, whereas RPT radionuclides are generally retained at target lesions in the body, allowing for more sustained prodrug activation (44,45). Cleavage by ^99m^Tc, ^111^In, and ^177^Lu roughly matched or outperformed efficiencies from EBRT. Different radionuclides showed different release efficiencies not directly related to linear energy transfer, suggesting that not all ionized molecules translate into radicals available for prodrug activation. Further work remains to dissect specific mechanisms. Our quenching studies indicate that free radical–driven linker cleavage mediates drug release, and our simulations suggest that a radionuclide’s ability to generate lower-energy electrons is more efficient for this process. We hypothesize that the efficacy of RAiDER may be a function of prolonged exposure to low-energy electron–generating ionizing radiation within a localized volume. Ionized molecules can combine with free electrons, and the generated radicals can recombine with nearby moieties depending on the local ionization concentrations. These events may explain the reduced drug release yield for highly localized ionizing radiations, such as α-emitters (46). Other factors, including tumor hypoxia or the intracellular distribution of RPT and prodrugs, may affect activation efficiency and biologic response. These interactions may also depend on the specific linker chemistry involved (21). Future studies characterizing the tumor microenvironment, together with localized prodrug activation, controlled experiments with EBRT, and molecular simulations, will further elucidate these mechanisms.

Heterogeneous tumor distribution is a barrier for antibodies, lipophilic drugs, and other targeted therapies. Incomplete drug distribution throughout the tumor can limit cytotoxicity and lead to treatment resistance (47). Antibody–drug conjugate linkers using enzymatic cleavage can be tuned to release their drug payload more efficiently across the tumor, including antibody–drug conjugates using MMAE (48). Although this leads to better tumor penetration by the drug payload, it can result in unwanted drug release elsewhere. Moreover, enzymatic cleavage strategies, such as with cathepsins, require intracellular prodrug uptake to endolysosomes, which may not always occur efficiently. Radionuclide activation does not have this requirement, which may expand the types of molecular targeting strategies adopted for RAiDER, for instance, by targeting the extracellular matrix or surface-expressed tumor antigens. It is also better suited for extensive metastases than EBRT.

Successful implementation of RAiDER will depend on the pharmacologic properties of each component. This may depend on cancer type and requires an understanding of the physiologic response to a combination of RPT and chemo- and immunotherapies. The optimal therapeutic index will likely be achieved with administration schemes that maximize tumor colocalization of RPT and prodrugs to effect highly localized prodrug activation. For agents tagged with a short-half-life nuclide, such as ^99m^Tc, this could be achieved by injecting macromolecular-conjugated prodrug to allow sufficient prodrug accumulation before radionuclide activation. Conversely, longer-half-life nuclides, such as ^177^Lu, can be injected before single or multiple prodrug doses to take advantage of prolonged tumor RPT retention (44). Choosing a radionuclide with higher drug release efficiency may not always be advantageous if a higher overall prodrug-activating radiation dose can be delivered to target tissues using other nuclides. The off-target effects of radionuclides should also be considered when choosing appropriate radionuclide–RAiDER combinations. RAiDER optimization would benefit from a systems pharmacology approach that combines experimental data and computation modeling. We have previously shown this approach to be helpful for other drug modalities (47).

Limitations of this study should be noted. We focused here on examining and maximizing tumor drug release using RAiDER. Although results show relatively negligible drug release in off-target sites, a better understanding of the factors guiding radiation–prodrug interactions in normal tissues is warranted. Furthermore, MMAE and exatecan may not be optimal for RAiDER. A broad palette of drugs can be used with radiation-labile linkers, and identifying those that synergize with RPT is essential. These include immunotherapies and therapies targeting protumorigenic signaling pathways activated by ionizing radiation (49). Systematic assessment of other radionuclides in vitro and in vivo is also needed to augment our understanding of the integrated physical, chemical, and biologic parameters that govern the efficacy of RAiDER.

## CONCLUSION

We have demonstrated that multiple clinically relevant radionuclides can control the spatiotemporal release of albumin-linked, radiation-cleavable prodrugs in vitro and in vivo. Judicious choice of radiopharmaceutical and caged-prodrug combinations enables an effective combination and systemic therapy approach with excellent therapeutic indices for disseminated disease. This work sets the stage for preclinical and clinical RAiDER evaluation across various malignancies.

## DISCLOSURE

The study was supported in part by the U.S. National Institutes of Health (DP2CA259675, DP2CA259675-01S1, R01GM138790, T32CA079443, U01CA206997, R21EB036323, R01DK097112, S10OD028499, R01CA187003, R00CA267560, R21CA279068, and R00EB030602), the U.S. Department of Defense (W81XWH-22-1-0061), the Lee Family Foundation, and the National Research Foundation of Korea (2022R1A6A3A03065564). Miles Miller has received unrelated support from Genentech/Roche, Pfizer, and Ionis Pharmaceuticals. Ralph Weissleder has consulted for Boston Scientific, ModeRNA, Earli, and Accure Health, none of which were involved in this research. Thomas Ng has received unrelated support from Lantheus and Bayer. Patents are pending or have been awarded to the authors and Massachusetts General Hospital. No other potential conflict of interest relevant to this article was reported.

## Notes

KEY POINTS **QUESTION:** Can radionuclides induce localized prodrug activation in vivo? **PERTINENT FINDINGS:** The ability of radionuclides to chemically activate prodrugs was demonstrated across clinically relevant dosings of radionuclides. Molecularly targeted radionuclides could induce active drug release from long-circulating prodrug conjugates preferentially in tumors over off-target sites in the body. Combination RPT with a model prodrug (caged MMAE) delayed tumor growth in an aggressive mouse model of anaplastic thyroid cancer without appreciable toxicity, whereas monotherapy did not. **IMPLICATIONS FOR PATIENT CARE:** The RAiDER strategy is feasible in vivo, paving the way to design effective combination radiopharmaceutical treatments with high therapeutic indices using this approach.
